# Glucose Oxidation to Pyruvate Is Not Essential for *Brucella suis* Biovar 5 Virulence in the Mouse Model

**DOI:** 10.3389/fmicb.2020.620049

**Published:** 2021-01-14

**Authors:** Leticia Lázaro-Antón, María Jesús de Miguel, Thibault Barbier, Raquel Conde-Álvarez, Pilar M. Muñoz, Jean Jacques Letesson, Maite Iriarte, Ignacio Moriyón, Amaia Zúñiga-Ripa

**Affiliations:** ^1^Department of Microbiology and Parasitology, Facultad de Medicina, ISTUN Instituto de Salud Tropical, University of Navarra, Pamplona, Spain; ^2^Navarra Institute for Health Research (IdiSNA), Pamplona, Spain; ^3^Unidad de Producción y Sanidad Animal, Centro de Investigación y Tecnología Agroalimentaria de Aragón (CITA), Zaragoza, Spain; ^4^Instituto Agroalimentario de Aragón-IA2, CITA-Universidad de Zaragoza, Zaragoza, Spain; ^5^Research Unit in Biology of Microorganisms (URBM), NARILIS, University of Namur, Namur, Belgium

**Keywords:** *Brucella*, metabolism, glucose, Entner-Doudoroff, virulence

## Abstract

*Brucella* species cause brucellosis, a worldwide extended zoonosis. The brucellae are related to free-living and plant-associated α2-*Proteobacteria* and, since they multiply within host cells, their metabolism probably reflects this adaptation. To investigate this, we used the rodent-associated *Brucella suis* biovar 5, which in contrast to the ruminant-associated *Brucella abortus* and *Brucella melitensis* and other *B. suis* biovars, is fast-growing and conserves the ancestral Entner-Doudoroff pathway (EDP) present in the plant-associated relatives. We constructed mutants in Edd (glucose-6-phosphate dehydratase; first EDP step), PpdK (pyruvate phosphate dikinase; phosphoenolpyruvate ⇌ pyruvate), and Pyk (pyruvate kinase; phosphoenolpyruvate → pyruvate). In a chemically defined medium with glucose as the only C source, the Edd mutant showed reduced growth rates and the triple Edd-PpdK-Pyk mutant did not grow. Moreover, the triple mutant was also unable to grow on ribose or xylose. Therefore, *B. suis* biovar 5 sugar catabolism proceeds through both the Pentose Phosphate shunt and EDP, and EDP absence and exclusive use of the shunt could explain at least in part the comparatively reduced growth rates of *B. melitensis* and *B. abortus*. The triple Edd-PpdK-Pyk mutant was not attenuated in mice. Thus, although an anabolic use is likely, this suggests that hexose/pentose catabolism to pyruvate is not essential for *B. suis* biovar 5 multiplication within host cells, a hypothesis consistent with the lack of classical glycolysis in all *Brucella* species and of EDP in *B. melitensis* and *B. abortus*. These results and those of previous works suggest that within cells, the brucellae use mostly 3 and 4 C substrates fed into anaplerotic pathways and only a limited supply of 5 and 6 C sugars, thus favoring the EDP loss observed in some species.

## Introduction

Members of the genus *Brucella* are α2-*Proteobacteria* that infect a wide range of vertebrates causing brucellosis in mammals (Al Dahouk et al., 2008; Whatmore, 2009; Soler-Lloréns et al., 2016), a zoonosis with a high impact on developing countries worldwide (McDermott et al., 2013). Although the genus includes an increasing number of species (Moreno, 2020), *Brucella abortus*, *Brucella melitensis*, and *Brucella suis* (often referred to as the classical smooth spp.) are by far those having the more severe impact on both livestock and humans and they were divided long ago into biovars following phenotypic criteria (Alton, 1987). Although for a long time thought to be a very homogeneous group (Hoyer and McCullough, 1968; Verger et al., 1987), the identification of new spp. and phylogenomic studies show that *B. abortus*, *B. melitensis*, *B. suis*, *Brucella neotomae*, *Brucella ovis*, and *Brucella canis* plus isolates from sea mammals and the common vole form a relatively heterogeneous core group separated from several early diverging brucellae (Wattam et al., 2014; Soler-Lloréns et al., 2016; Moreno, 2020). These studies also show that, while all *B. abortus* and *B. melitensis* biovars group into two clades, the five *B. suis* biovars show a greater diversity that is inconsistent with their current taxonomic status as a single sp. (Moreno and Moriyón, 2002; Al Dahouk et al., 2008; Scholz et al., 2008a; Whatmore, 2009; Moreno, 2020).

**FIGURE 1:**
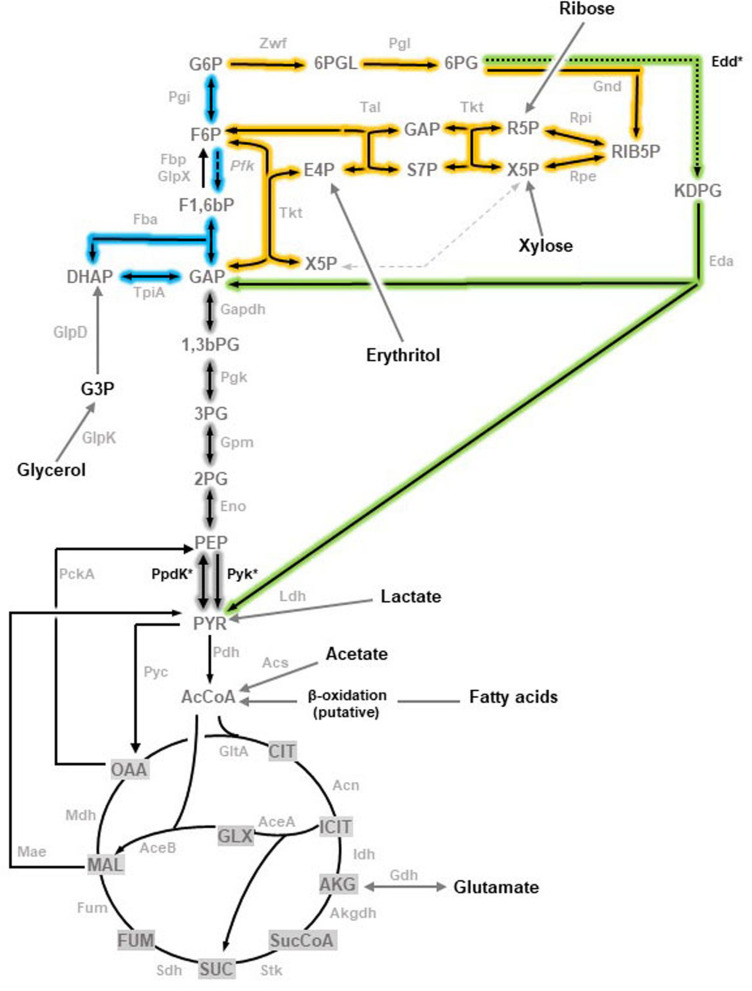
Topology of the three glycolytic routes in *Brucella* (adapted from Zúñiga-Ripa et al., 2018). Embden-Meyerhof-Parnas (EMP) pathway, often referred to as glycolysis, is shown in blue with a dashed arrow marking the reaction catalyzed by the key enzyme Pfk absent in brucellae. Pentose Phosphate (PP) pathway is complete and highlighted in yellow. The Entner-Doudoroff (ED) pathway is shown in green with a dotted arrow marking the step catalyzed by Edd. Reactions that are shared by the three pathways are highlighted in gray. The enzymes corresponding to the genes investigated are marked with and asterisk. Abbreviations used are Metabolites: 1,3,bPG, 1,3-bisphosphoglycerate; KDPG, 2-keto-3-deoxy-phosphogluconate; 2PG, 2-phosphoglycerate; 3PG, 3-phosphoglycerate; 6PGL, 6-P- (gluconolactone; 6PG, 6-phosphogluconate; AcCoA, acetyl-coenzyme A; AKG, alpha-ketoglutarate; CIT, citrate; ICIT, isocitrate; DHAP, dihydroxyacetone-P; E4P, erythrose-4-P; F1,6bP, fructose-1,6-bisphosphate; F6P, fructose-6-P; FUM, fumarate; G6P, glucose-6-P; GAP, glyceraldehyde-3-P; G3P, glycerol-3-P; GLX, glyoxylate; MAL, malate; OAA, oxaloacetate; PEP, phosphoenolpyruvate; PYR, pyruvate; R5P, ribose-5-P; RIB5P, ribulose-5-P; S7P, sedoheptulose-7-P; SUC, succinate; SucCoA, succinyl-coenzyme A; X5P, xylulose-5-P. Enzymes: Edd, 6-phospho-D-gluconate dehydratase; Gnd, 6-phosphogluconate dehydrogenase; Pgl,6-phosphogluconolactonase; Acs, acetyl-coenzyme A synthetase; Acn, aconitate hydratase; Akgdh, alpha-ketoglutarate dehydrogenase; GltA, citrate synthase; Eno, enolase; Fbp, GlpX, fructose-1,6-bisphosphatase; Fba, fructose bisphosphate aldolase; Fum, fumarase; Zwf, glucose-6-P dehydrogenase; Pgi, glucose-6-P isomerase; Gdh, glutamate dehydrogenase; Gapdh, glyceraldehyde-3-P dehydrogenase; GlpD, glycerol-3-P dehydrogenase; GlpK, glycerol kinase; Idh, isocitrate dehydrogenase; AceA, isocitrate lyase; Eda, 2-dehydro-3-deoxy-phosphogluconate aldolase; Ldh, lactate dehydrogenase; Mdh, malate dehydrogenase; AceB, malate synthase; Mae, malic enzyme; PckA, phosphoenolpyruvate carboxykinase; Pfk, phosphofructokinase; Pgk, phosphoglycerate kinase; Gpm, phosphoglycerate mutase; Pyc, pyruvate carboxylase; Pdh, pyruvate dehydrogenase; Pyk, pyruvate kinase; PpdK, pyruvate phosphate dikinase; Rpi, ribose-5-phosphate isomerase; Rpe, ribulose-5-P-3-epimerase; Sdh, succinate dehydrogenase; Stk, succinyl-coenzyme A synthetase; Tal, transaldolase; Tkt, transketolase; Tpi, triose P isomerase.)

The weight of the evidence shows that the brucellae have evolved from environmental α2-*Proteobacteria* (Moreno, 2020). Since they are facultative intracellular pathogens unable to persist in nature outside their hosts, this origin implies that they have probably adapted their metabolism to the peculiarities of the *Brucella* containing vacuoles (BCV) where they multiply. Undoubtedly because of their early identification and greater impact on domestic livestock and humans, metabolism has been investigated almost exclusively in *B. abortus, B. melitensis*, and biovars 1 and 3 of *B. suis*. These spp. and biovars, although auxotrophic only for a few vitamins and occasionally for a few amino acids (Gerhardt and Wilson, 1948; Plommet, 1991), are often described as fastidious because of their complex requirements for primary isolation (peptone-yeast extract media, often supplemented with serum) and slow growth. However, *B. suis* biovar 5 and *Brucella microti*, both rodent-associated brucellae, display much faster growth (Scholz et al., 2008b; Zúñiga-Ripa et al., 2018), which suggests a more ancestral metabolism. Consistent with this, we have recently found (Machelart et al., 2020) that, like the environmental α2-*Proteobacteria* neighbors, the Entner-Doudoroff pathway (EDP) is fully active in those rodent-associated spp. so that glucose is fueled into the tricarboxylic acid cycle (TCA) mostly through EDP with little contribution of the Pentose Phosphate Pathway (PPP). Thus, whereas *B. suis* biovar 5 mutants in *edd* (coding for the 6-phosphogluconate dehydratase involved in the first step of EDP) have a severe growth defect, mutation of *gnd* (6-phosphogluconate dehydrogenase of the first step of PPP) has only a reduced effect. On the other hand, *B. abortus, B. melitensis*, and *B. suis* other than biovar 5 rely exclusively on PPP because all carry a disabling mutation in *edd*. This shows the dispensability of EDD in the spp. that cause disease in livestock and, consistent with the lack of the phosphofructokinase of the Embden-Meyerhof-Parnas (classical glycolysis) in all brucellae (Barbier et al., 2018), supports the hypothesis that glucose fueling into TCA is not essential in BCVs and was thus lost in some clades. Although *B. suis* biovar 5 and *B. microti* represent a suitable mode to test this hypothesis, we have consistently failed to obtain a double *edd*-*gnd* mutant, in all likelihood because PPP becomes essential when EDD is not functional (Machelart et al., 2020). However, as that hypothesis can be tested by blocking pyruvate synthesis at other levels of the central C pathways, in this work we applied this approach by deleting *edd* in *B. suis* biovar 5 and, instead of *gnd*, the genes putatively coding for pyruvate phosphate dikinase (*ppdK*) and pyruvate kinase (*pyk*) (Figure 1). Here, we present experiments that confirm the corresponding predicted metabolic phenotypes as well as the results of an assessment of virulence in the mouse model of brucellosis.

## Materials and Methods

### Bacterial Strains and Growth Conditions

The bacterial strains and plasmids used are listed in Supplementary Table 1. At the University of Navarra, bacteria were routinely grown in peptone-glucose [TSB, bioMerieux; bio-Trypcase (17 g/L), bio-Soyase (3 g/L), glucose (2.5 g/L), NaCl (5 g/L), and K_2_HPO_4_ (2.5 g/L)], or on this medium supplemented with agar (TSA). For the animal experiments at CITA (see below), the inocula were grown on Blood Agar Base No. 2 [BAB2, Oxoid; proteose peptone (15 g/L), liver digest (2.5 g/L), yeast extract (5 g/L), NaCl (5 g/L), and agar (12 g/L)] a basal medium previously shown to be equivalent to TSA with a large number of strains (in preliminary experiments, it was confirmed that the strains used in this study grew similarly on both media, as expected) (De Miguel et al., 2011). To study the phenotype of the metabolic mutants, a base medium [modified Plommet’s medium; (Plommet, 1991; Barbier, 2014)] was used: 9.2 g/L K_2_HPO_4_; 3.0 g/L KH_2_PO_4_; 0.1 g/L Na_2_S_2_O_3_; 5.0 g/L NaCl; 0.2 g/L nicotinic acid; 0.2 g/L thiamine; 0.07 g/L pantothenic acid; 0.5 g/L (NH_4_)_2_SO_4_; 0.01 g/L MgSO_4_; 0.1 mg/L MnSO_4_; 0.1 mg/L FeSO_4_; 0.1 mg/L biotin. For metabolic studies, this base medium was supplemented with the appropriate C source at 1 g/L. When necessary, peptone-glucose was supplemented with kanamycin (50 μg/ml), polymyxin B (2 μg/ml), or sucrose (5%). All strains were stored in skimmed milk (Scharlau) at −80°C.

### Growth Curves

The following protocol was used to avoid any nutrient carry over. Bacteria were first grown in 10 ml of peptone-glucose in a 50 ml flask at 37°C for 18 h with orbital agitation, harvested by centrifugation, and then resuspended in 10 ml of base medium supplemented with the appropriate C source at an optical density of 0.1 at 600 nm (O.D._600__nm_). This broth was incubated with orbital agitation at 37°C for 18 h, cells harvested by centrifugation, resuspended to an O.D._600__nm_ of 0.1 in 1 ml of the same medium, and transferred to Bioscreen plates (200 μl/well) in technical triplicates. Growth was monitored every 0.5 h at 420−580 nm with continuous shaking at 37°C in a Bioscreen C incubator (Lab Systems) using wells with sterile medium as the blank. All experiments were repeated at least three times.

### DNA Manipulations

Genomic sequences of *B. suis* 513 (the reference strain of the *B. suis* biovar 5) were obtained from the Kyoto Encyclopedia of Genes and Genomes (KEGG) database^1^. Searches for DNA and protein homologies were carried out using the National Center for Biotechnology Information (NCBI)^2^, the European Molecular Biology Laboratory (EMBL)-European Bioinformatics Institute server^3^, and The Broad Institute of Harvard and MIT-*Brucella* group databases. Primers were synthesized by the Sigma-Genosys (Haverhill, United Kingdom). Restriction-modification enzymes were used as recommended by the manufacturers. Plasmid DNAs were extracted with the QIAprep Spin Miniprep (Qiagen GmbH, Hilden, Germany) and genomic DNAs from individual colonies by boiling in water. When needed, DNA was purified from agarose gels using the QIAquick Gel Extraction Kit (Qiagen).

### Mutagenesis

The *B. suis* 513 *Bs5*Δ*ppdK* in-frame mutant in *ppdK* [described in a previous work (Zúñiga-Ripa et al., 2018)] carried a deletion encompassing 86% of the corresponding gene. To obtain *Bs5*Δ*pyk*, a first plasmid (pAZI-36; Supplementary Table 1) was prepared as follows. First, two PCR fragments were generated using oligonucleotides Pyk-F1 (5′- GCTGACGTCGCGCTATTATT-3′) and Pyk-R2 (5′-CGTGGCGAGAATCTTGACC-3′), which amplified a 282 bp fragment including codons 1–12 of *pyk*, as well as 246 bp upstream of the *pyk* start codon; and oligonucleotides Pyk-F3 (5′-GGTGCAAGATTCTCGCCACGGGTGCAACCAATATGCTGC-3′) and Pyk-R4 (5′-CGCTCTGAATTCGCATTTG-3′), which amplified a 296 bp fragment including the last 60 bp of *pyk*. To join the two fragments, a third PCR used oligonucleotides Pyk-F1 and Pyk-R4 for amplification and the complementary regions between Pyk-R2 and Pyk-F3 for overlapping. The resulting fragment, containing the *pyk* deletion lacking 96% of the wild-type ORF, was cloned into pCR2.1 (Invitrogen) to obtain pAZI-36. After sequence verification, the deletion allele was excised using *Bam*HI–*Not*I and cloned into a pJQKm suicide vector (Scupham and Triplett, 1997). The resulting pLZI-1 mutator plasmid (Supplementary Table 1) was transformed into *E. coli* strains TOP10F’ and S17λpir and transferred to *B*. *suis* 513 by conjugation. Integration of the suicide vector in the chromosome was selected by polymyxin (*B. suis* 513 is intrinsically resistant) and kanamycin resistance, and excision of pLZI-1 (producing *Bs5*Δ*pyk* by allelic exchange) by polymyxin and sucrose resistance and kanamycin sensitivity. The resulting colonies were screened by PCR with primers Pyk-F1 and Pyk-R4, which amplified a fragment of 578 bp and 1917 bp in the mutant and parental strain, respectively.

*Bs5*Δ*ppdK*Δ*pyk* was constructed introducing pLZI-1 into *Bs5*Δ*ppdK* by conjugation and selection by polymyxin and sucrose resistance and kanamycin sensitivity, and confirmed by PCR using oligonucleotides Pyk-F1 and Pyk-R4. To check for both mutations, the internal primer Pyk-R5 (5′- TTTTCCGTCATCGATCAACA -3′) hybridizing in the deleted region was used.

To construct mutant *Bs5Δedd*, the suicide mutator plasmid pNPTSΔ*edd* (Supplementary Table 1), carrying the *edd* deleted allele (Machelart et al., 2020) was introduced into *E. coli* S17λpir by transformation. Then, pNPTSΔ*edd* was introduced into *B. suis* 513 by conjugation. Following the protocol described above, colonies from the second recombination were screened by PCR with primers Edd-F1 (5′-GGCACGATTTCATCAGCGCA-3′) and Edd-R4 (5′-CCGCCATTCATGGCATTCTGG-3′), which amplified a fragment of 1,447 bp in the mutant and a fragment of 3,271 bp in the parental strain. The deletion removed 56% of the ORF and was identified using the internal primer Edd-R5 (5′-TCCTGAATGCGTTTATGTGC-3′) which hybridized in the deleted region.

To construct *Bs5*Δ*pyk*Δ*edd, Bs5*Δ*ppdK*Δ*edd*, and *Bs5*Δ*ppdKΔpykΔedd*, pNPTSΔ*edd* was introduced into *Bs5*Δ*pyk, Bs5*Δ*ppdK*, and *Bs5*Δ*ppdKΔpyk* by conjugation and primers Edd-F1, Edd-R4, and Edd-R5 were used to screen the resulting colonies.

### Virulence Assays in Mice

Seven-week-old female BALB/c mice (Envigo-Harlan Laboratories, Barcelona, Spain) were accommodated under BSL-3 biosafety containment conditions in the facility of Centro de Investigación y Tecnología Agroalimentaria de Aragón (CITA; Registration code ES502970012025) with water and food *ad libitum*. The animal handling and procedures were in accordance with the current European (directive 86/609/EEC) and Spanish (RD/53/2013) legislation and authorized by the Animal Welfare Committee of the institution. For each strain, inoculum was prepared from a 24 h culture on Blood Agar Base No. 2 (see above “Bacterial Strains and Growth Conditions”) at 37°C. Bacterial suspensions in sterile phosphate buffered saline (0.85% NaCl, 0.1% KH_2_PO_4_, 0.2% K_2_HPO_4_; pH 6.85) were spectrophotometrically adjusted to 1 × 10^9^ colony forming units (CFU) and diluted to the required concentration. Mice (*n* = 5) were inoculated intraperitoneally with approximately 1 × 10^5^ CFU in 0.1 mL of the corresponding inoculum (exact doses were retrospectively assessed by CFU accounts on BAB plates) and then euthanized 2 and 8 weeks after inoculation. Spleens were aseptically removed, individually weighed, homogenized in nine volumes of sterile saline buffer and serial 10-fold dilutions plated by triplicate on BAB plates for CFU accounts. The identity of the isolates was confirmed by PCR. Individual data (mean CFU/spleen) were normalized by logarithmic transformation and the mean log CFU/spleen values and the standard deviation (*n* = 5) calculated for statistical comparisons by one-way ANOVA followed by the Dunnett’s test.

## Results

### The Simultaneous Deletion of *edd*, *ppdK*, and *pyk* Abolishes *B. suis* 513 Growth on 5 and 6 C Sugars

Whereas the oxidative PPP yields phosphoenolpyruvate (PEP) that is then converted into pyruvate, the EDP produces PEP and directly pyruvate (Figure 1). Since pyruvate can be converted directly into acetyl-CoA to feed the TCA, when glucose is the only C source it can be predicted: (i) that the steps connecting PEP and pyruvate should be dispensable for growth if EDP is active; and (ii) that an *edd* mutant defective in these steps should not grow on glucose.

**FIGURE 2:**
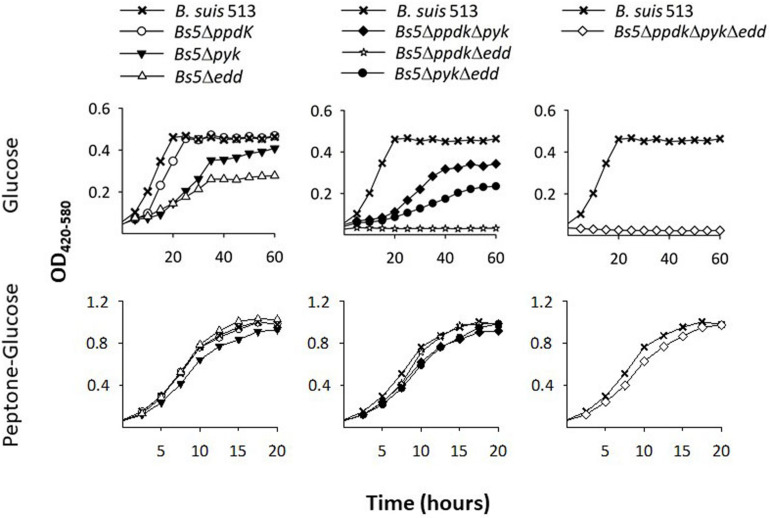
Deletion of *ppdk*, *pyk*, and *edd* abrogates growth of *B. suis* 513 on glucose as sole C source. Growth curves were obtained in modified base Plommet’s medium supplemented with glucose **(upper panels)** or in peptone-glucose broths **(lower panels)**. Each point represents the mean ± standard error of an experiment performed in technical triplicates (error bars are within the size of the symbols). The experiments were repeated at least three times with similar results.

According to genomic predictions, the *B. suis* 513 (the reference strain of the *B. suis* biovar 5) enzymes involved in PEP-pyruvate conversions would be a (putative) pyruvate phosphate dikinase (PpdK) and a (putative) pyruvate kinase (Pyk) (Figure 1). Therefore, as a first test for those predictions, we examined *Bs5*Δ*ppdK* and *Bs5*Δ*pyk* for growth on glucose and on peptone-glucose as a control. As can be seen in the upper left panel of Figure 2, *Bs5*Δ*ppdK* grew on glucose reaching the level of the parental strain with a short delay and similar generation times (about 8 h during the exponential phase). On glucose, although *Bs5*Δ*pyk* displayed a longer generation time (about 11 h), it also reached a stationary phase level like that of the parental strain (Figure 2, upper left panel). Both mutants grew normally in peptone-glucose (Figure 2, lower left panel). As a control, we included a mutant in *edd*. Whereas this *Bs5*Δ*edd* mutant grew normally on peptone-glucose (Figure 2, lower left panel), we observed that it grew less and more slowly than its parental *B. suis* 513 strain on glucose (Figure 2, upper left panel). This result confirms the functionally of the EDP and, since growth was not abrogated, it also shows a minor activity of a complementary glucose oxidative route, which should be the oxidative PPP because of the lack of phosphofructokinase and hence classical glycolysis in all brucellae.

Based on these results, we then constructed and tested the double *Bs5*Δ*ppdK*Δ*pyk* and triple *Bs5*Δ*ppdK*Δ*pyk*Δ*edd* mutants. We found that, while growth of the double mutant *Bs5*Δ*ppdK*Δ*pyk* was delayed but not arrested on glucose (Figure 2, upper central panel), *edd* became essential for growth when both *ppdK* and *pyk* were mutated (Figure 2, upper right panel). On the other hand, these mutants grew normally in complex medium (Figure 2, lower central right panels). These results strongly suggest that PpdK and Pyk are functional, confirm that *B. suis* 513 has an operative ED route and are consistent with our predictions.

In the experiments presented thus far, we noticed that *Bs5*Δ*ppdK* grew faster than *Bs5*Δ*pyk*, which implies that *ppdK* cannot fully replace *pyk* when the bacteria are growing on glucose (Figure 2, upper left panel). This prompted us to investigate whether the reactions catalyzed by these two enzymes are similarly effective when combined with the EDP. For this, we compared mutants *Bs5*Δ*ppdK*Δ*edd* and *Bs5*Δ*pyk*Δ*edd* on glucose. Unexpectedly, the results (Figure 2, upper central panel) showed no growth for *Bs5*Δ*ppdK*Δ*edd* and generation times for *Bs5*Δ*pyk*Δ*edd* not very different from those of the *Bs5*Δ*edd* single mutant, suggesting a major and not dispensable role for PpdK. No growth defect was apparent on peptone-glucose (Figure 2, lower central panel).

**FIGURE 3:**
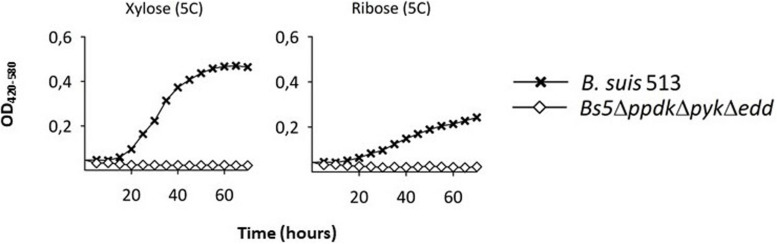
A triple *Bs5*Δ*ppdk*Δ*pyk*Δ*edd* mutant cannot grow on xylose or ribose. Each point represents the mean ± standard error of an experiment performed in technical triplicates (error bars are within the size of the symbols). The experiment was repeated at least three times with similar results.

We also noticed that *Bs5*Δ*edd* showed longer generation times and stationary phase yields lower than those of *Bs5*Δ*ppdK* or *Bs5*Δ*pyk* (Figure 2, left panel). As indicated above, growth of *Bs5*Δ*edd* under these conditions should occur only through the oxidative PPP, being in this regard similar to the three classical smooth *Brucella* spp. The PPP yields PEP through glyceraldehyde-3-P (GAP), and then PEP yields pyruvate through the Pyk and PpdK catalyzed reactions (Figure 1). Therefore, a direct proof of PPP as the only sugar catabolic route remaining in mutant *Bs5*Δ*edd* would be that Pyk and Ppdk become essential for growth on pentoses when EDP is not functional. We confirmed this prediction (Figure 3) by taking advantage of the ability of *B*. *suis* 513 to grow on xylose and ribose as the only C source (Zúñiga-Ripa et al., 2018).

### The Simultaneous Deletion of *edd*, *ppdK*, and *pyk* Does Not Affect *B. suis* 513 Virulence in Mice

**FIGURE 4:**
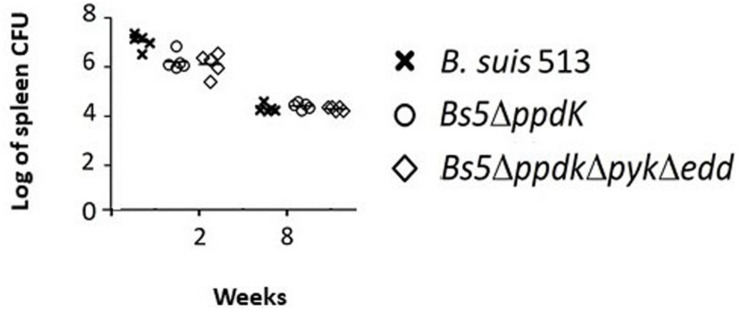
The triple mutant *Bs5*Δ*ppdk*Δ*pyk*Δ*edd* is not attenuated in the mouse model. Each point is the mean ± standard deviation (*n* = 5) of the logs of CFUs per spleen in technical triplicates. There were no statistical differences at any of the two times tested (one-way ANOVA followed by Dunnett’s test; *p* > 0.5).

The phenotype of the *Bs5*Δ*ppdK*Δ*pyk*Δ*edd* provided a tool to investigate whether the catabolism of 6 (and 5 C) sugars was essential during infection. To investigate this, we inoculated BALB/c mice with *Bs5*Δ*ppdK*Δ*pyk*Δ*edd* and, as controls, *Bs5*Δ*ppdK* and *B. suis* 513, and determined the CFU/spleen after 2 or 8 weeks (acute and chronic phase of infection, respectively). We found that the triple mutant *Bs5*Δ*ppdK*Δ*pyk*Δ*edd* was not attenuated in this virulence model (Figure 4).

## Discussion

In this work, we confirm and extend our previous results supporting the existence of an active ED route in *B. suis* 513 (biovar 5) that together with the oxidative PPP sustains growth of this biovar on glucose as the only C source *in vitro* (Machelart et al., 2020). In keeping with the prediction that these routes produce PEP/pyruvate, we also found that growth requires PpdK and Pyk, the former apparently playing a major and non-dispensable role *in vitro*. Indeed, simultaneous dysfunction of Edd, PpdK, and Pyk also abolished the ability of *B. suis* 513 to grow on xylose or ribose. Indirectly, the data also confirm the lack of an active EMP pathway, consistent with the absence of phosphofructokinase in all brucellae (Barbier et al., 2018). It has to be noted that but for the *ppdK* one (Zúñiga-Ripa et al., 2018), the mutants investigated were not complemented despite several attempts (Lázaro-Antón, Moriyón and Zúñiga-Ripa; unpublished results). We have already detected this experimental difficulty with some *Brucella* mutants affected in intermediary C pathways, and this could be due to the intricacies of metabolic regulatory loops, plasmid stability, and other factors (Zúñiga-Ripa et al., 2014). However, while strict proof would require such experiments, it has to be stressed that the phenotype of the mutants studied here fully correspond with the predicted ones, which strongly suggests that the conclusions that can be drawn are valid. Also worth commenting is that, while further research is necessary to ascertain the metabolic peculiarities of the slow- and fast-growing brucellae, the observation that deletion of *edd* considerably reduces the growth rates of *B. suis* 513 suggests that the shift from EDD to PPP as the major route for sugar metabolism could be one of the reasons for these different phenotypes.

On the connection virulence-metabolism in brucellae, here we examined whether glucose oxidation beyond pyruvate is necessary for *B. suis* 513 virulence in the mouse model, and we obtained a negative answer. Considering the *in vitro* phenotypes of *B. suis* 513 and its *Bs5*Δ*ppdK*Δ*pyk*Δ*edd* mutant, this conclusion can be extended to at least xylose and ribose, two pentoses feeding into the PPP. As discussed below, these conclusions do not exclude other uses of hexoses and/or pentoses by the brucellae.

Several works offer insight on the role of hexose/pentose metabolism in *Brucella* virulence. A signature-tagged mutagenesis screening in mice identified a *gluP* [glucose/galactose transporter; (Essenberg et al., 1997)] mutant of *B. abortus* 2308 among those attenuated 8 weeks after infection but not among those identified as attenuated at 2 weeks post-infection (Hong et al., 2000). This mutant, however, was not critically compromised [virulent/*gluP* mutant co-infection competitive index at week 8 23.4 as compared to 72.4 for a *gltD* (glutamate synthase) mutant tested in parallel]. In subsequent work with *gluP*, Xavier et al. (2013) proposed that an increased glucose availability mediated by peroxisome proliferator-activated receptor γ (PPARγ) facilitates *B. abortus* 2308 survival during the chronic phase in alternative activated macrophages. Although other authors have interpreted these results to mean that glycolysis may play an important role in metabolism and virulence of intracellular *Brucella* (Gao et al., 2016), the multiplication of *B. abortus* in mouse spleens occurs early during infection [when no role for *gluP* was observed (Hong et al., 2000)] before the numbers of bacteria reach a short plateau after which it decreases progressively (Grilló et al., 2012). Therefore, the *gluP* studies suggest that glucose or galactose are used after the acute phase of infection for purposes other than major C/energy sources for multiplication in at least those laboratory models. Similar considerations can explain the mild attenuation of *B. suis* 1330 ribose kinase (*rbsk*) and 6-phosphogluconate dehydrogenase (*gnd*) Tn5 mutants observed in macrophage-like human THP-1 cells 48 h after infection (log CFU reduction for both mutants of 1.8 vs. 5 for genes involved in amino acid synthesis) (Köhler et al., 2002). Moreover, in our hands a *B. suis* 1330 mutant in *gnd* is severely attenuated (Machelart et al., 2020). On the other hand, other works that provide results on how virulence is affected by mutation of enzymes of hexose/pentose metabolism cannot be unequivocally interpreted in terms of metabolism. For example, a 3 log CFU attenuation was found for a *B. suis* 1330 P-glucose isomerase (*pgi*) Tn5 mutant (Foulongne et al., 2000) but, indeed, this mutation has pleiotropic effects, including that on the synthesis of mannose and hexosamine, two sugars required for lipopolysaccharide building. Similarly, a glucose-6-P dehydrogenase (*zwf*) mutant of *B. abortus* 544 was described as completely unable to multiply in Hela cells but surprisingly the mutant was severely hampered in invasiveness (Kim et al., 2003), a phenotype that strongly suggests defects not related to its ability to multiply within cells.

Whereas our results are not in open conflict with those summarized in the previous paragraph, the lack of a role in virulence of *pyk* and *ppdK* manifested in the phenotype in mice of *Bs5*Δ*ppdK*Δ*pyk*Δ*edd* apparently contradicts conclusions obtained in other studies. Gao et al. (2016) constructed a *B. abortus* S2308 *pyk* mutant that, in contrast to the parental strain, was impaired for growth on glucose but not on pyruvate. This *B. abortus pyk* mutant did not multiply in RAW 264.7 macrophages and was attenuated in BALB/c mice (approximately 2.5 log CFU less than the parental strain 1 and 5 weeks after infection). More recently, Pitzer et al. (2018) reported that a *B. abortus* 2308 *pyk* mutant proved to be defective in the activity of Pyk displayed reduced ability to metabolize glucose, fructose, and galactose but not ribose, xylose, arabinose or erythritol, and was attenuated in C57BL/6 mice. The reasons for the discrepancies in both attenuation and the range of substrates used *in vitro* are not obvious. For the attenuation, a plausible explanation would be that these studies have been conducted in *B. abortus* 2308 and ours with *B. suis* 513. As emphasized in the Introduction, *B. suis* 513 (but not *B. abortus* 2308) is fast-growing and can use a wider menu of substrates as the only source of C and energy including lactate and glutamate, which by themselves do not support growth of *B. abortus* 2308W (Zúñiga-Ripa et al., 2018). In the host, these abilities of *B. suis* 513 could provide a way to circumvent the PEP → pyruvate conversion as lactate can provide pyruvate, and the TCA cycle can also be fed by glutamate (Zúñiga-Ripa et al., 2018). Discrepancies in the use of C sources *in vitro* by *B. abortus* in different works could be explained by subtle differences between strains 2308 and 2308W, as they are not genetically identical (Suárez-Esquivel et al., 2016) and/or by the experimental conditions. Gao et al. (2016) tested 2308 growth in a medium containing glucose or pyruvate and mineral salts but also 0.1% yeast extract, which makes the medium non-minimal and could thus account for the differences in growth on glucose of 2308 and 2308W. Also, the utilization of hexoses and pentoses by 2308 in the work of Pitzer et al. (2018) was tested in a Biolog system, which implies an undefined medium and, therefore, provides no information on the use of those substrates as the only C/energy sources. Like in the study of Gao et al. (2016) the minimal medium used by Pitzer et al. (2018) contained 0.1% yeast extract.

Regarding *ppdK*, we reported in a previous work that this gene is necessary for *B. abortus* 2308W virulence (Zúñiga-Ripa et al., 2014), and this was confirmed in the study of Pitzer et al. (2018). Recently, we showed that the homologous *ppdK* mutant in *B. suis* 513 was not attenuated in mice and we elucidated the reasons for this discrepancy: this *B. suis* biovar 5 strain can use PpdK and PEP carboxykinase (PckA) for PEP synthesis *in vitro* (Figure 1), PckA catalyzing oxaloacetate conversion into PEP, while *B. abortus* 2308W uses only PpdK, that catalyzes the PEP-pyruvate interconversion (Zúñiga-Ripa et al., 2018). Indeed, we showed that *B. suis* 513 attenuation occurs in the double PckA-PpdK mutant (Zúñiga-Ripa et al., 2018).

Consistent with the absence of phosphofructokinase (Pfk) in all brucellae and the dispensability of the EDP, our results are in line with the hypothesis that hexose/pentose catabolism through the TCA is not necessary for growth in BCVs, which may account for the loss of EDP in *B. abortus*, *B. melitensis*, and some *B. suis* biovars. As PpdK catalyzes an amphibolic reaction that can support the synthesis of 3 to 6 C biosynthetic precursors and Pyk is catabolic, this could explain why the former seems to have a more important role for growth of at least *B. suis* 513. The data presented here together with those of previous works are consistent with a model (Zúñiga-Ripa et al., 2014, 2018) in which the brucellae thrive intracellularly by using 3 and 4 C substrates with a limited supply of 5 and 6 C sugars that are devoted to biosynthesis.

## Data Availability Statement

The raw data supporting the conclusions of this article will be made available by the authors, without undue reservation.

## Ethics Statement

The animal study was reviewed and approved by the Animal Welfare Committee of CITA.

## Author Contributions

AZ-R, JL, MI, and IM conceived and coordinated the study. LL-A and AZ-R carried out the genomic analyses, mutants construction, and metabolic tests. MM, TB, RC-Á, and PM contributed in mutant construction, growth measurements, and experiments in mice. AZ-R, LL-A, and IM wrote the manuscript. All authors analyzed the results and approved the manuscript content.

## Conflict of Interest

The authors declare that the research was conducted in the absence of any commercial or financial relationships that could be construed as a potential conflict of interest.

## Footnotes

1 http://www.genome.jp/kegg/

2 http://www.ncbi.nlm.nih.gov/

3 http://www.ebi.ac.uk/
