# Supplementary material for: Glucose Oxidation to Pyruvate Is Not Essential for Brucella suis Biovar 5 Virulence in the Mouse Model
Source: Front Microbiol. 2021 Jan 14;11:620049. doi: 10.3389/fmicb.2020.620049 (PMC7840955; doi:10.3389/fmicb.2020.620049)
Supplement: Supplementary file 1 [file Table_1.DOCX]

| Table S1. Bacterial strains and plasmids | | |
| --- | --- | --- |
| Strain or plasmid | Characteristics^1 ,2, 3^ | Reference/Source |
| *Brucella* |  |  |
| *B. suis* 513 | Wild type, biovar 5 reference strain | (Vershilova et al., 1983) |
| Bs5ΔppdK | *B. suis* 513 *ppdK*_Δ35-888_ | (Zúñiga-Ripa et al., 2018) |
| Bs5Δpyk | *B. suis* 513 *pyk*_Δ36-1375_ | This work |
| Bs5ΔppdKΔpyk | *B. suis* 513 *ppdK*_Δ35-888_*pyk*_Δ36-1375_ | This work |
| Bs5Δedd | *B. suis* 513 *edd*_Δ35-1326_ | This work |
| Bs5ΔppdKΔedd  Bs5ΔpykΔedd | *B. suis* 513 *ppdK*_Δ35-888_*edd*_Δ35-1326_  *B. suis* 513 *pyk*_Δ36-1375_*edd*_Δ35-1326_ | This work  This work |
| Bs5ΔppdKΔpykΔedd | *B. suis* 513 *ppdK*_Δ35-888_*pyk*_Δ36-1375_*edd*_Δ35-1326_ | This work |
| *Escherichia coli* |  |  |
| *E. coli* S17λpir | Mating strain with plasmid RP4 inserted into the chromosome (Tpr Smr *rec*A thi hsdRM+, lambda pir phage lysogen RP4::2-Tc::Mu::Km Tn7) | (Simon et al., 1983; Miller and Mekalanos, 1988) |
| *E. coli* TOP10F´ | F ^-^ lacIq Tn 10 (Tetr) *mcrA* Δ(mrr-hsdRMS-mcrBC) 80lacZΔM15 ΔlacX74 *recA*1*ala*D139 Δ (ara-leu)7697 *galU galK rpsL* endA1 *nupG* | Invitrogen |
| Plasmid |  |  |
| pCR2.1-TOPO | Cloning vector, Km^R^ | Invitrogen |
| pJQKm | Suicide vector, Km^R^, Sac^s^ | (Scupham and Triplett, 1997) |
| pNPTS | Suicide vector, Km^R^, Sac^s^ | (Spratt et al., 1986) |
| pNPTSΔ*edd* | 1447 bp containing the *edd* (BAB2_0458) deletion allele, generated by PCR and cloned into pNPTS | (Machelart et al., 2020) |
| pAZI-36 | 578 bp containing the *pyk* (BAB1_1761)^3^ deletion allele, generated by PCR and cloned into pCR2.1-TOPO | This work |
| pLZI-1 | *BamH*I*/Not*I fragment from pCR2.1-TOPO-*BAB*Δ*pyk* cloned into de corresponding site of pJQKm | This work |
| ^1^ Nucleotides deleted are in sub index characters. | | |
| ^2^ Km, kanamycin; Sac, sucrose.  ^3^ Since the genome of *B. suis*513 is not annotated, the locus numbers of the orthologue genes in *B. abortus* 2308W are indicated: *edd* (BAB2_0458) and *pyk* (BAB1_1761). In addition, the locus number corresponding to *ppdK* is BAB1_0525. | | |
